# Exploring the role of group support models for HIV prevention among adolescent girls and young women (AGYW) in sub–Saharan Africa (SSA): a scoping review

**DOI:** 10.3389/frph.2026.1892424

**Published:** 2026-07-27

**Authors:** Faith Mary Musvipwa, Buhle Lubuzo, Mosa Julia Letsielo, Roisin Elizabeth Drysdale, Elmari Briedenhann, Catherine Elizabeth Martin, Saiqa Mullick

**Affiliations:** Wits RHI, Faculty of Health Sciences, University of Witwatersrand, Johannesburg, South Africa

**Keywords:** adolescent girls and young women (AGYW), group support models, hiv prevention, pre exposure prophylaxis (PrEP), sub-Saharan Africa (SSA)

## Abstract

**Background:**

Adolescent girls and young women (AGYW) remain disproportionately affected by HIV, particularly in sub-Saharan Africa (SSA), where most AGYW living with HIV reside. Although group-based support interventions have been introduced to improve HIV prevention outcomes, evidence on how these models are structured and their effectiveness for AGYW remains limited. This scoping review mapped and synthesised evidence on group support interventions for HIV prevention among AGYW in SSA, with a focus on pre-exposure prophylaxis (PrEP) uptake, adherence, and retention in care.

**Methods:**

A scoping review was conducted between September and November 2024 following established methodological guidance. Group support models were defined as structured or semi-structured interventions that bring AGYW together to provide peer support, HIV and sexual and reproductive health education, psychosocial support, and skills-building to improve HIV prevention outcomes. Peer-reviewed qualitative, quantitative, and mixed-methods studies were searched across four databases using keywords. Grey literature was excluded. Data were charted and analysed thematically to map and synthesize evidence on group support interventions.

**Results:**

The initial search yielded 1,901 results after 62 duplicates were removed. 40 papers were eligible. Following title/abstract and full-text screening, 11 studies met the inclusion criteria. Full-text articles were excluded because they did not meet the target population criteria (*n* = 5), lacked focus on group support models (*n* = 12), were irrelevant to study outcomes (*n* = 10), or were not behavioural/health-related studies (*n* = 2). Group-based HIV prevention models commonly included peer-led support, HIV and sexual and reproductive health education, psychosocial support, mentorship or buddy systems, communication and negotiation skills-building, and PrEP adherence discussions. Across studies, these interventions were reportedly associated with improved HIV prevention knowledge, increased PrEP uptake and early adherence, stronger motivation to remain engaged in care, reduced stigma, improved social support, and greater confidence in sexual health decision-making. The findings suggest that group support models may help address social, cultural, and gender-related barriers to HIV prevention by creating supportive and empowering spaces for AGYW. However, evidence on long-term PrEP adherence, retention in care, scalability, sustainability, cost-effectiveness, and comparative effectiveness of different approaches was limited. Few studies reported objective adherence outcomes or longer-term HIV prevention impacts.

## 1 Background

In 2023, 39.9 million people globally were living with human immunodeficiency virus (HIV), with 1.3 million (1–1.7 million) newly acquired infections reported that year (1). Although women and girls accounted for only 46% of new infections worldwide, in sub-Saharan Africa (SSA), they accounted for 62%, highlighting a significant disparity in this region (1). Among the most vulnerable populations globally were adolescent girls and young women (AGYW) aged 15–24 years, with 4,000 new HIV infections each week (1). Notably, 3,100 of these acquisitions occurred in SSA (1), underscoring the region's disproportionate burden. The high incidence of HIV among AGYW underscores the crucial need for effective HIV prevention strategies. Pre-exposure prophylaxis (PrEP) has emerged as a key preventive strategy in the battle against HIV (2). While PrEP has demonstrated efficacy, challenges related to ongoing effective use and retention in prevention persist (3). For PrEP to be effective, consistent, effective use during periods of exposure is fundamental (3), yet many AGYW struggle with maintaining regular use (4). This difficulty is attributed to various factors, including socioeconomic barriers, stigma, lack of support, gender-based violence, and depression (5–7). Within this context, group-based support models have been developed and implemented specifically for AGYW to address these intersecting barriers. The focus of this review is therefore on group support interventions designed for AGYW within HIV prevention and care programmes, particularly those aimed at improving PrEP uptake, adherence, and retention in SSA.

HIV among African women and girls is driven by a complex interplay of gender inequalities that intersect across individual, cultural, economic, and systemic levels (8, 9). AGYW in SSA face unique vulnerabilities, including socio-economic disadvantages, gender inequality, and cultural norms that exacerbate their risk of HIV acquisition (9, 10). With the rollout of long-acting HIV prevention options, such as injectable PrEP, there is growing potential to expand prevention choices and improve uptake among more vulnerable populations. However, many young women may still prefer daily oral PrEP due to familiarity or individual lifestyle considerations. Regardless of the prevention method selected, sustained engagement in HIV prevention services, including adherence support, follow-up care, and integrated sexual and reproductive health services, will remain critical to ensuring optimal protection and long-term effectiveness. Group support models are structured, facilitated interventions that bring together individuals of similar age, gender, or social background to participate in regular group sessions focused on HIV prevention. Unlike individual counselling or routine clinical care, these models emphasise peer interaction, shared learning, psychosocial support, and facilitated discussions to encourage HIV prevention behaviours. Depending on the intervention, they may include HIV and PrEP education, skills building, adherence support, risk reduction counselling, and linkage to sexual and reproductive health services, to improve PrEP uptake, adherence, and retention (11–13). Group support models have been proposed to improve PrEP retention (14). Evidence from peer-led models in other health interventions, particularly antiretroviral therapy (ART) adherence clubs, demonstrates that group-based approaches can improve retention in care, strengthen treatment adherence, and enhance patient empowerment through shared experiences and collective accountability (15, 16). Additionally, emerging literature suggests that support groups may offer important mental health benefits, including reduced social isolation, decreased stigma, and improved emotional well-being, which are critical considerations given the substantial mental health burden among populations at elevated risk for HIV (15, 16). Together, these broader health and psychosocial benefits further strengthen the rationale for group support models as a multifaceted strategy for HIV prevention.

Peer-led approaches, mostly for AGYW, have been widely endorsed as strategies for sexual health education, health promotion, and HIV prevention for the past two decades, even though there has been limited empirical evidence of their efficacy in changing behaviour (12, 16). Given this gap, highlighting and mapping these models can provide insights into the range of existing strategies, identify common components, and gaps. Furthermore, available studies often vary in how group support models are defined, structured, and implemented, making it difficult to determine which components are most impactful and scalable within resource-constrained settings in SSA. This lack of clarity creates an important evidence gap for policymakers and program implementers seeking to optimise HIV prevention strategies for AGYW. In addition, most existing reviews have focused broadly on HIV prevention or PrEP adherence interventions without isolating group-based support models for AGYW, and few have systematically mapped the specific intervention components and delivery characteristics that were reported across different contexts. This scoping review aims to (1) identify and describe the key components and implementation approaches of group-based HIV prevention models for AGYW in SSA, and (2) map and synthesise reported evidence on group support interventions related to HIV prevention use and retention.

## 2 Methods

### 2.1 Study design

This review followed the Joanna Briggs Institute (JBI) methodological guidance for scoping reviews, using the Population, Concept, and Context (PCC) framework by Peters et al. (17) to guide the development of the research question, inclusion criteria, and search strategy. The PCC framework was applied to ensure that all relevant dimensions of the review question were captured and to check for any potentially missed inclusion or exclusion criteria during protocol development. (See Supplementary Appendix Table A2 search terms based on the PCC framework). For a full search strategy adapted for each database, see Supplementary Appendix Table A1 (Search strategy and databases). We adhered to the principles outlined in the Preferred Reporting Items for Systematic Reviews and Meta-Analyses (PRISMA) Extension Guidelines for Scoping Reviews (PRISMA-ScR) (18). The presentation of results is guided by four of the PRISMA's five essential stages. The stages consist of identifying relevant studies; study selection; charting the data; and collating, summarising, and reporting results. However, we did not include the consultation exercise in this review, which involves the use of stakeholder consultations in scoping reviews (19).

Stage 1: The eligibility criteria

### 2.2 Inclusion criteria

Only original research available in full text and published in English between 2012 and 2024 was included. This time frame was selected to capture contemporary evidence on HIV prevention approaches following the emergence and early scale-up of PrEP around 2012. The review included quantitative, qualitative, and mixed-methods study designs. Studies were included if they reported evidence on group support models for HIV prevention among AGYW aged 15–24 years. Studies that included participants beyond this age range (e.g., 15–30 years) were eligible only if findings specific to AGYW could be extracted or were clearly reported. Similarly, studies were included if they involved both males and females but presented disaggregated data or findings relevant to AGYW. Studies focusing exclusively on males or that did not report results separately for AGYW were excluded. However, two studies that deviated from the stipulated age range were included because their findings were highly relevant to the experiences and support needs of AGYW and contributed valuable insights into the functioning and reported outcomes of group support models. Studies conducted within educational institutions, healthcare, or community settings were included. Healthcare settings included clinics and other formal health service points, while community settings encompassed schools, youth centers, local gathering spaces, and other non-clinical venues.

The following principles were used to determine the studies that met the inclusion criteria:

1. Studies conducted in SSA.
2. Studies reporting on group support models for HIV prevention broadly, among AGYW aged 15–24 years.
3. Studies published from 2012 to 2024
4. Studies conducted within educational institutions, healthcare, or community settings.

Studies with the following characteristics were excluded:

1. Studies that were not conducted in SSA.
2. Studies not including HIV prevention among AGYW aged 15–24 years.
3. Studies conducted outside educational institutions, healthcare, or community settings, such as laboratory-based studies, modelling or simulation studies, editorial/commentary papers, and secondary analyses not reporting primary intervention data within real-world implementation contexts.

Stage 2: Identifying Relevant Studies

Systematic searches for the identification of relevant studies for inclusion in this review started with electronic searches conducted through reputable databases. Our search was conducted in four databases representing various health disciplines: PubMed, Scopus, Web of Science, and Cinahl. We conducted searches on Google Scholar using the citation tracking function and checked for papers that had cited the papers included in this review. In addition, we hand-searched the bibliographies of all included studies for additional articles that we may have missed in our original search and found an additional two articles. We did not search for grey literature as we were interested in original research.

### 2.3 The search management

Following the rigorous and intensive searches, all retrieved articles recorded from the electronic databases and hand searches deemed to meet the inclusion criteria were then imported to EndNote (version 20). Moreover, the EndNote virtual library was created specifically for this study to manage records and data throughout the review and remove duplicates of the same records.

Stage 3: Study selection

Search results were imported into EndNote, and duplicates were removed. The initial screening process was conducted by two reviewers (FMM and MJL), where titles and abstracts were screened, and those that did not meet the inclusion criteria were excluded. Pertinent abstracts, of which there was uncertainty about inclusion status, were moved to full-text screening for articles to be fully examined for eligibility. Both reviewers screened full-text articles for final inclusion using a standardized eligibility screening form. The results of the screening were discussed with another reviewer (BL), who assessed the full text of the remaining articles, and disagreements were resolved through discussions.

For this review, we included two papers that deviated from the stipulated age group due to the scarcity of data. These were the only studies identified that were outside the age range but provided relevant insights into group support models for HIV prevention among AGYW. The first study included very young adolescents aged 12–14 years (20), while the second included adolescents and youth aged 12–19 years (21). Given the scarcity of data specific to the target age group, these studies helped capture a broader understanding of the topic, offering valuable context and highlighting potential gaps in the literature. In terms of synthesis, the findings from these two studies were not analysed as a separate subgroup but were instead integrated into the overall thematic synthesis. Additionally, the papers offered relevance to key themes and therefore provided critical insights and unique perspectives that align with our review's core objectives; hence, they were worth including, even though the age group differs. All other studies that included participants outside the age range were excluded if data specific to AGYW could not be disaggregated.

Stage 4: Charting the data

Data extraction was conducted independently by two reviewers (FMM and MJL), and discrepancies were resolved through discussion with the third reviewer (BL) to ensure consistency. Extracted data were then collated and synthesized to identify patterns, trends, and gaps in literature relevant to group support models for HIV prevention among AGYW. We extracted details such as authors, publication year, study method, population characteristics, model, structure, content, delivery methods, study setting, and key outcomes. After extracting the information, the data were then systematically charted into a pre-designed data extraction table (See Supplementary Appendix Table A3: characteristics of studies included) that established a clear and concise summary of the extracted data, which was then analysed and interpreted. For this stage of the review, articles were read several times, and a thematic coding scheme was developed inductively through discussion by the reviewers (FMM, BL, and MJL).

### 2.4 Assessment of methodological quality

In this review, no formal quality assessment was performed, as the objective was to map the existing literature rather than evaluate the quality of individual studies. The absence of formal quality appraisal was a deliberate methodological decision aligned with the review objectives and design. The inclusion of the two studies outside the target age range may have broadened the synthesis.

## 3 Results

### 3.1 Search results

We retrieved a total of 1,901 results after duplicates (62) were removed. Following the initial screening of all the titles and abstracts, 40 papers were eligible. To expand our search, we also searched the bibliographies of the papers that matched the inclusion criteria, and an additional two were found. Full-text articles were excluded from the scoping review for several reasons. Five articles did not align with the target population, while twelve were excluded due to their study focus not meeting the inclusion criteria. Additionally, ten articles were removed because their reported study outcomes were not relevant, and two were excluded as they primarily focused on behavioural or other health-related topics rather than the specific scope of the review. A total of eleven articles were included in the final analysis (See Supplementary Appendix Figure A1 flow of searches).

### 3.2 Characteristics of the included articles

The reviewed studies examined group support models for HIV prevention and retention among AGYW. Although the inclusion criteria targeted HIV prevention broadly, most interventions incorporated a PrEP component or involved participants who used or were eligible for PrEP, ensuring relevance to biomedical prevention. Participants' characteristics in the support groups included a diverse age range, predominantly 12–25 25 years (22, 26). Recruitment was primarily community-based, with some studies also engaging youth in educational and clinical settings (21, 27). Study methods were predominantly mixed, including both qualitative and quantitative approaches (21, 22, 27, 29). Facilitation approaches also differed across models, with some groups led by trained peers to enhance relatability and trust, while others were facilitated by healthcare providers, counsellors, or trained mentors to ensure clinical accuracy and structured guidance. In several models, group support was directly linked to PrEP education and provision, offering referrals, on-site initiation, or adherence support; however, in other cases, the connection to PrEP services was less explicit, focusing more broadly on sexual health and risk reduction. This variation highlights differing levels of integration between group-based support and biomedical prevention services. In healthcare settings, support was often integrated into clinic visits or offered through mobile outreach services, while community-based groups were delivered in youth centers, schools, or other peer-support venues.

The number of participants in the studies evaluated varied, ranging from around 20 to larger groups exceeding 100 per study. Across the eleven studies, a total of approximately 1,000 participants were involved. Six studies were conducted in South Africa (22, 25–29), two in Uganda (30), two in Malawi (20, 21), one in Tanzania (22), one in Kenya, one in Liberia (27) and two in Zimbabwe (24, 29), with some studies conducted in multiple locations. Papers were published between 2020 and 2023, highlighting the recent focus on HIV prevention support and its impact on AGYW in SSA *(See* Supplementary Appendix Table A3 *characteristics of studies included).*

### 3.3 Overview of study designs and methods

Across the included studies, research designs included purely qualitative interviews (*n* = 2) (20, 22), mixed methods (*n* = 5) (24–30), quantitative methods (*n* = 4) (21, 27–29). Data collection included semi-structured interviews, attendance logs, pre/post surveys, drug level assays, SMS/phone check-ins, and WhatsApp engagement metrics. Analyses ranged from thematic coding of qualitative data to descriptive and inferential statistics assessing PrEP uptake, adherence, and intervention feasibility.

### 3.4 Types of group support models

A diverse range of group support models were identified across the included studies, tailored to meet the unique needs of AGYW in different settings. To facilitate interpretation of the diverse interventions identified in this review, group support models were classified into four broad categories based on their primary focus, delivery approach, and support mechanisms: (I) Peer-Led Clubs and Empowerment Groups; (II) PrEP Adherence and Support Clubs; (III) Hybrid and Digitally Enhanced Models; and (IV) Community-Integrated Support Structures. Despite their diversity, these models shared a common aim of fostering engagement, empowerment, and HIV prevention through structured peer support.

I. Peer-led clubs and empowerment groups:

Four (4) studies described peer-led clubs that focused on building self-efficacy and empowerment among AGYW (22). These groups followed a structured curriculum delivered by trained peer mentors or facilitators and covered topics such as life skills, sexual and reproductive health (SRH), gender norms, and HIV prevention (22, 25). For example, the “Empowerment and Livelihood for Adolescents (ELA)” youth clubs in Uganda combined SRH education with vocational skills training such as soap and menstrual pad making, reinforcing both economic and health empowerment (20). Peer-led empowerment clubs created safe spaces where young women unpacked gender norms, practiced goal-setting, and built self-efficacy through structured activities and open discussion (20, 22).

II. PrEP adherence and support clubs:

Another prominent model was the PrEP adherence support club, specifically designed to improve initiation, continuation, and adherence to PrEP among AGYW (25, 26, 30). These clubs provided safe, youth-friendly environments where participants could share experiences, discuss challenges such as stigma and disclosure, and receive ongoing counselling, including drug-level feedback sessions (20, 22, 24, 28–30). In some cases, monthly or quarterly gatherings served both social and educational purposes, helping to normalize PrEP use among young women.

III. Hybrid and digitally enhanced models:

Innovative digital models were also observed (26, 30). The “PrEP Smart” intervention used moderated WhatsApp groups led by facilitators and PrEP ambassadors to create a virtual support network (26). These digital platforms were used to share information, moderate discussions on SRH and PrEP, and build peer support remotely (26, 30). SMS reminders and digital check-ins were also used to boost retention and engagement (26, 30).

IV. Community-integrated support structures:

Many models were delivered in community-based settings such as schools and local gathering spaces (20, 22, 27, 28). Integrating support groups into familiar environments enhanced accessibility and fostered trust among participants (20, 24). The Determined, Resilient, Empowered, AIDS-free, Mentored and Safe (DREAMS) initiative is a PEPFAR-funded programme that uses a multi-sectoral approach to prevent HIV/AIDS among AGYW and their male sex partners (MSPs). In several cases, group interventions were embedded within larger programs like DREAMS, which provided additional layers of support, including health services, education support, and social asset building (24).

### 3.5 Core features of group support interventions for AGYW

Across the included studies, several key features emerged as central to the design and implementation of group-based HIV prevention interventions focused on AGYW. These elements contributed to how support models were perceived, received, and sustained in various SSA settings.

I. Psychosocial empowerment

Group interventions consistently aimed to enhance AGYW's confidence, autonomy, and ability to navigate social and relationship dynamics. Sessions frequently included topics on gender-based violence, healthy relationships, communication skills, and future planning (22, 24). These activities helped AGYW reflect on personal challenges, gain a sense of agency, and build self-esteem, crucial for making informed decisions about HIV prevention and overall well-being (22, 24).

II. Integrated sexual & reproductive health education

SRH education was a central component across most models. Curricula addressed puberty, pregnancy, family planning, HIV/sexually transmitted infections (STIs), and PrEP, often tailored to the age and knowledge levels of participants (20, 21, 27). Notably, several models intentionally incorporated the integration of SRH content with life skills development and, in some cases, vocational training. This multidimensional approach emerged as a prominent feature across a few group support models, reflecting a shift from narrowly focused HIV prevention messaging towards more comprehensive empowerment strategies. By addressing broader social and economic vulnerabilities alongside health education, these integrated models aimed to strengthen agency, support sustained engagement in prevention services, and enhance overall well-being among AGYW. The integration of SRH topics with life skills and vocational training created a holistic learning experience, empowering AGYW with both knowledge and practical skills (21, 28).

III. Peer-led facilitation & mentorship

Many interventions relied on peer facilitators or mentors, often slightly older women with shared life experiences, to deliver content and guide discussions (20, 22, 24–26). This approach created safe, relatable spaces that fostered trust and encouraged open dialogue (20, 22). Some models also incorporated “PrEP ambassadors” or mentor figures who supported participants both online and in person (26).

IV. Delivery format & structure

The format and duration of sessions varied widely, from monthly 90-minute empowerment club sessions to more frequent (22, 25, 28), intensive interventions delivered three times a week (22). Several studies included unstructured or participant-led sessions to enhance flexibility and responsiveness to AGYW's evolving needs (22). Digital components, such as WhatsApp groups and SMS reminders, complemented in-person sessions in some models (24, 26, 30).

V. Settings & target groups

Programs adapted content, delivery, and structure to the specific age, schooling status, and setting of AGYW (22). The settings of the HIV prevention group support models, ranging from clinical to community spaces such as schools and universities, were designed to enhance accessibility and meet AGYW where they are. The diversity in settings allows for a more inclusive approach, ensuring that AGYW from various socioeconomic backgrounds, educational levels, and geographical locations can participate in the groups (22, 25). Group support models were implemented across diverse venues, including clinics, schools and universities, and community centres, allowing programmes to reach AGYW in settings that were both accessible and contextually appropriate (20, 22). Some studies also described intentional age segmentation within these models, tailoring content to participants' developmental stages (20). Very young adolescents (12–14 years) were typically engaged through simpler, school-friendly modules focused on foundational sexual health knowledge, while older AGYW (16–25 years) participated in more in-depth discussions addressing topics such as PrEP disclosure, relationship dynamics, and autonomy in prevention decision-making (24). This approach highlights the importance of age-responsive programming in enhancing relevance, engagement, and potential effectiveness of group-based HIV prevention interventions (20). Support group models in the reviewed studies demonstrate context-specific adaptations to improve accessibility and participation. In urban settings, inner-city university students typically engage in campus-based empowerment clubs, whereas village-based youth attend community-centred Empowerment and Livelihood for Adolescents (ELA) sessions. Similarly, programmes accommodate differing educational statuses by aligning meetings with school timetables while also offering weekend or after-hours sessions to ensure inclusion of adolescents who are out of formal education (21).

### 3.6 Pathways to impact for HIV prevention and PrEP retention

The group support models reported contributed to HIV prevention among AGYW through several interlinked pathways. These included enhancing awareness and knowledge, building motivation for PrEP uptake and continued use, and addressing barriers to effective use of PrEP.

I. HIV knowledge and vulnerability perception

Many interventions strengthened AGYW's understanding of HIV transmission, prevention, and the role of PrEP. Sessions often demystified misconceptions around PrEP, addressed side effects, and clarified the benefits of consistent use (24, 29, 30). This educational component played a foundational role in increasing awareness and shaping positive attitudes toward HIV prevention tools.

II. Motivation and behavioural support for PrEP retention

Beyond knowledge, models included motivational elements designed to support continued PrEP use. Group support models offered regular check-ins, peer encouragement, and behavioural reinforcement (24, 29, 30). Digital features like SMS reminders and WhatsApp group discussions provided ongoing engagement and motivation outside of formal sessions (26, 30).

III. Social support and normalisation

Peer-driven formats helped normalize PrEP use and reduce stigma. Participants often reported feeling more comfortable discussing sensitive topics within groups where others were facing similar challenges (22, 25, 30). The group setting also served as a buffer against misinformation and social pressures, offering validation and solidarity (22, 26, 30).

IV. Addressing structural and relational barriers

Some interventions directly tackled gender power imbalances, intimate partner dynamics, and social expectations that deter HIV prevention behaviours. By incorporating modules on gender norms, control in relationships, and GBV, group support sessions empowered AGYW to negotiate safer sexual practices and make autonomous health decisions (22).

## 4 Discussion

This scoping review mapped existing literature on group support models for HIV prevention among AGYW in SSA. Our findings suggest that group support interventions incorporating peer facilitation, psychosocial empowerment, and interactive discussions were generally associated with improved PrEP engagement and adherence (22, 31). These approaches may represent pathways to foster trust, create safe spaces to discuss sensitive issues, reduce stigma, and strengthen participants' confidence and motivation to use PrEP consistently (20, 22). These findings suggest that the active components of successful group support models extend beyond the provision of HIV prevention information. Opportunities for peer interaction, shared lived experiences, ongoing encouragement, and practical problem-solving may help participants overcome common barriers to PrEP use, including stigma, fear of disclosure, low vulnerability perception, and difficulties maintaining adherence. Interventions that combined psychosocial support with practical adherence strategies, therefore, appeared better positioned to influence sustained PrEP engagement than education-only approaches (22, 27, 30). In contrast, interventions that primarily focused on information provision without opportunities for peer interaction or ongoing support appeared less likely to address the social and behavioural barriers influencing PrEP use. However, the diversity of intervention designs, delivery settings, outcome measures, and study methodologies limits direct comparison between models and makes it difficult to identify a single optimal approach. Much of the existing evidence remains heterogeneous, with relatively few rigorously evaluated group support interventions specifically designed for PrEP users. The limited evidence base is also partly explained by considerable variation in content, intensity, duration, facilitator training, and outcome measures, making comparisons across studies difficult. In addition, many group support models were evaluated using small-scale pilot or feasibility studies, limiting the strength and generalisability of the evidence. Future research should prioritise robust comparative evaluations to determine which group support models' components are essential for improving PrEP uptake, adherence, and persistence among AGYW across different contexts. This highlights the need for further implementation research to identify the most effective group support components, optimal delivery strategies, and approaches that can be adapted across diverse settings. The findings showed that most of the evidence on this topic comes from South Africa, likely reflecting the country's high HIV burden and early PrEP rollout. The paucity of evidence in other SSA countries, particularly those in Western and Central Africa, may be due to policy barriers relating to HIV testing, health systems, and HIV prevention (32). This review highlights the multifaceted landscape of HIV prevention and retention strategies for AGYW across SSA. Our review identified key themes shaping the role of group support models in HIV prevention among AGYW in SSA. These include strategies to improve PrEP retention, leveraging peer networks for motivation and social support, and fostering empowerment and life skills to enhance health decision-making (25). Group support models are designed not only to deliver information about HIV prevention and PrEP but also to encourage consistent use through regular engagement, accountability, and motivation. Some studies showed that repeated reinforcement through regular meetings and follow-ups was essential for continued group engagement, suggesting that retention in HIV prevention is not solely a medical concern but also a behavioural one that requires ongoing support (33). Motivation plays a significant role in the emotional and psychological aspects of PrEP use (33), where participants are encouraged to see their health as a priority and are reassured about the benefits of PrEP. The findings suggest that AGYW benefitted from integration of health education, group support sessions, text and WhatsApp messaging, and general counselling delivered in youth-friendly clinics. Where reported, these models appeared to support PrEP uptake and persistence, highlighting the potential of multi-component interventions in promoting HIV prevention among AGYW. Evidence on the effectiveness of peer-based support groups in improving PrEP retention remains limited, with some studies reporting no measurable impact on sustained use (34). The limited impact on retention may reflect aspects of how the models were implemented, such as session frequency or content, as well as limitations in study design, including small sample sizes or non-randomized methods. While group-based approaches show promise in strengthening engagement in HIV prevention, their effectiveness and scalability may be influenced by contextual and structural factors, such as health system capacity, resource availability, and local implementation contexts. In addition, limited HIV and PrEP knowledge among adults (21) (20 years and older), together with adherence challenges associated with PrEP methods that require daily dosing, such as oral PrEP, may reduce the overall effectiveness of these interventions (25). The authors noted the need for further research, particularly larger randomized clinical trials, to assess the effectiveness of these peer-based interventions in improving PrEP retention. Nevertheless, these models were generally found to be acceptable to participants and offered additional benefits. For example, they created supportive spaces for AGYW to share practical strategies for managing side effects, foster peer encouragement, and build social networks that may strengthen engagement in HIV prevention services more broadly. While the direct effect on retention is unclear, these findings suggest that group support models may contribute to psychosocial outcomes that are important for long-term prevention behaviours.

Addressing gender norms and stigma, delivering comprehensive health education, and ensuring accessibility in diverse settings were also crucial. The inclusion of family planning and STI education empowers AGYW to take control of their sexual health beyond HIV prevention, making the program more comprehensive and relevant to their lives.

Empowerment-based curricula played a key role in challenging harmful gender norms, giving AGYW the tools to make informed decisions and assert their rights, including sexual and reproductive rights. Furthermore, curricula that focused on challenging traditional gender roles and providing AGYW with the skills to negotiate sexual decisions were essential for fostering greater agency and reducing the risk of HIV (22, 24, 35). The review highlights that HIV prevention support groups must be adaptable to local needs, which means tailoring content to the specific challenges and concerns of AGYW in each region, reflecting a strong awareness of the cultural and regional diversity across SSA. This cultural sensitivity is crucial for ensuring the effectiveness of these programs across different countries.

Content on intimate partner violence (IPV) emerged as a critical component of support group sessions, given the well-established links between IPV and increased HIV vulnerability, reduced relationship power, and limited ability to negotiate safer sex practices (36). Exposure to IPV can also undermine consistent PrEP use by limiting autonomy in health decision-making and creating barriers to accessing prevention services. Incorporating IPV-related content within group models helps participants recognise unhealthy relationship dynamics, strengthen safety planning, and access appropriate support services. AGYW perceived group support as beneficial for fostering a sense of community, providing emotional support, and building confidence to address barriers such as IPV and stigma.

AGYW were able to share their experiences with stigma and learn skills around PrEP disclosure during sessions (36). Peer-led support also enhanced trust and comfort, making it easier for AGYW to discuss sensitive topics like HIV prevention, sexual health, and retention on PrEP (16). This dynamic is important in environments where stigma and silence about sexual health issues are prevalent (37).

Clinical settings provide access to healthcare professionals and resources, while community-based settings offer a more familiar and informal environment for learning and discussion. The combination of both settings ensures that health education and support are integrated into the everyday lives of AGYW, making it easier for them to access and continue with PrEP (5).

Evidence on group support models for HIV prevention among AGYW in SSA is largely concentrated in South Africa, with limited research from other regions likely reflecting policy and health system constraints. Overall, these models integrate peer support, empowerment, and health education to promote PrEP uptake and persistence, recognising that retention is both behavioural and psychosocial. While measurable effects on PrEP retention remain inconclusive, group models were widely acceptable and contributed to motivation, social connectedness, and coping strategies that may support longer-term prevention behaviours. Programs that addressed gender norms, stigma, IPV, and broader sexual and reproductive health needs strengthened AGYW's agency and decision-making, underscoring the value of comprehensive and culturally responsive curricula. Finally, delivering support across both clinical and community settings enhanced accessibility and relevance, highlighting the importance of adaptable, context-sensitive approaches to HIV prevention.

### 4.1 Implications

The findings of this scoping review suggest that group support models have potential as a complementary strategy for strengthening HIV prevention among AGYW, particularly by addressing psychosocial, structural, and behavioural drivers of PrEP use. However, the limited and geographically concentrated evidence base highlights the need for more rigorous research across diverse SSA contexts to better determine effectiveness, especially for PrEP retention. Future programs should prioritise culturally responsive, empowerment-focused curricula that integrate sexual and reproductive health, address gender norms and IPV, and leverage peer support. Policymakers and implementers should also consider multi-component and flexible delivery approaches across clinical and community platforms to enhance accessibility, engagement, and sustainability of HIV prevention services.

## 5 Conclusion

This scoping review shows that group-based HIV prevention models for AGYW in SSA commonly combine peer support, empowerment approaches, structured health education, and culturally responsive strategies to encourage PrEP uptake and engagement in HIV prevention services. Across the included studies, these models were associated with reported improvements in HIV prevention knowledge, motivation, and early engagement with services. However, important evidence gaps remain. Few studies explored long-term PrEP adherence, persistence, or retention in care, and there was limited evidence comparing different group support approaches, their implementation, scalability, or sustainability. In addition, outcomes related to sustained PrEP use, HIV incidence reduction, and cost-effectiveness were rarely reported. Future research should focus on rigorously evaluating specific group-based models, particularly peer-led and adherence-focused interventions, to better understand which components drive the strongest and most sustainable outcomes. More evidence on long-term retention, objective adherence measures, acceptability, scalability, and cost-effectiveness will be critical to guide the optimisation and scale-up of effective HIV prevention programmes for AGYW in SSA.

### 5.1 Strengths and limitations

This review comprehensively mapped existing group support models by including both quantitative and qualitative studies and extracting data on group models, structure, content, delivery methods, setting, and location. The results are not without limitations. Although an extensive search strategy was used across four databases, supplemented by manual searching of reference lists and citation tracking using Google Scholar, only a small number of studies met the inclusion criteria, with just two additional papers identified through supplementary searching. This reflects a scarcity of empirical research on group support models for HIV prevention among AGYW in SSA, despite the importance of such models for improving engagement with PrEP and other HIV prevention strategies. This limited evidence base is partly due to the relatively recent rollout of PrEP, with most studies emerging post-2020, and the fact that many group-based interventions are embedded within broader programmes such as DREAMS and may not be reported in peer-reviewed literature. The review is further limited by the exclusion of grey literature and non-English publications, which may have omitted relevant programmatic evidence from organisations such as USAID, PEPFAR, and UNAIDS, where community-based interventions are often implemented but not always published in academic journals. In addition, heterogeneity across studies in terms of intervention design, populations, settings, outcomes, and measurement of adherence limited comparability and synthesis. Many studies also relied on short-term or self-reported outcomes, with limited evidence on long-term follow-up, scalability, and cost-effectiveness. This review maps available evidence but does not establish effectiveness or causality, particularly given the variability in study designs and contexts. This is further constrained by the absence of a formal methodological quality appraisal, which limits the ability to assess the strength and certainty of the evidence. Furthermore, although the review focused on AGYW aged 15–24 years, some included studies involved broader age groups; while relevant data were extracted where possible, this may affect the specificity of conclusions for the target population. Despite these limitations, the review provides a structured synthesis of existing group support models for HIV prevention among AGYW in SSA, highlighting key features, gaps, and areas for future research to inform the development of more contextually appropriate and scalable interventions.
