# Supplementary material for: Exploring the role of group support models for HIV prevention among adolescent girls and young women (AGYW) in sub–Saharan Africa (SSA): a scoping review
Source: Front Reprod Health. 2026 Jul 27;8:1892424. doi: 10.3389/frph.2026.1892424 (PMC13454023; doi:10.3389/frph.2026.1892424)
Supplement: Supplementary file 1 [file Table1.docx]

**Appendix 1**

List of figures and tables

Table 1 search strategy and databases

| **Database** | **Search strategy** |
| --- | --- |
| PubMed/ MEDLINE | (("adolescent girls"[All Fields] OR "young women"[All Fields] OR "AGYW"[All Fields] OR "15-24 years"[All Fields] OR "youth"[All Fields]) AND ("group service"[All Fields] OR "support service"[All Fields] OR "clubs"[All Fields] OR "HIV prevention"[All Fields]) AND ("sub-Saharan Africa"[All Fields] OR "SSA"[All Fields] OR "Africa"[All Fields] OR "cultural factors"[All Fields] OR "socio-economic factors"[All Fields] OR "geographical factors"[All Fields])) AND (2012:2024[pdat])  Search date: 9 September 2024  Limits applied:  Publication years: 2012–2024  Language: English  Original research articles only |
|  |  |
|  |  |
|  |  |
| Scopus | TITLE-ABS-KEY (("Adolescent girls" OR "young women" OR "AGYW" OR "15-24 years" OR youth) AND ("Group service" OR "support service" OR clubs OR "HIV prevention") AND ("Sub-Saharan Africa" OR SSA OR Africa OR "cultural factors" OR "socio-economic factors" OR "geographical factors"))  Search date: 8 October 2024  Limits applied:  Publication years: 2012–2024  Language: English  Document type: Original research articles  Subject areas: Medicine, Health Sciences, Social Sciences, and Multidisciplinary Studies |
| Web of Science | ((ALL= (Adolescent girls OR young women OR AGYW OR 15-24 years OR youth)) AND ALL= (Group service OR support service OR clubs OR HIV prevention)) AND ALL=(Sub-Saharan Africa OR SSA OR Africa OR cultural factors OR socio-economic factors OR geographical factors)  Search date: 24 September 2024  Limits applied:  Publication years: 2012–2024  Language: English  Document type: Original research articles |
| Cinahl | ("adolescent girls" OR "young women" OR "AGYW" OR "15-24 years" OR youth) AND ("group support" OR "support service" OR clubs OR "HIV prevention") AND ("sub-Saharan Africa" OR SSA OR Africa OR "cultural factors" OR "socio-economic factors" OR "geographical factors")  Search date: 13 November 2024  Limits applied:  Publication years: 2012–2024  Language: English  Publication type: Original research articles |

Table 2 search terms based on the PCC framework

| Population | Adolescent girls OR young women OR AGYW OR 15-24 years OR youth |
| --- | --- |
| Concept | Group service OR support service OR clubs OR HIV prevention |
| Context | Sub-Saharan Africa OR SSA OR Africa OR cultural factors OR socio-economic factors OR geographical factors |

*Figure 1 flow of searches*

Studies relevant by full text

(n = 11)

**Included**

**Eligibility**

PUBMED (n = 662)

**Search Terms**

**Set 1:** Adolescent girls, young women, AGYW, 15-24 years, youth

**Set 2:** Group service OR support service OR clubs OR HIV prevention

**Set 3:** Sub-Saharan Africa OR SSA OR Africa OR cultural factors OR socio-economic factors OR geographical factors

**Screening**

Duplicates removed (n = 62)

**Identification**

SCOPUS (n = 850)

WEB OF SCIENCE (n = 396)

CINAHL (n = 55)

Total records identified through database search

(n = 1963)

Studies relevant by title and abstract

(n = 40)

**Identification of studies via databases**

Records excluded (n = 1861)

Records screened

(n = 1901)

Full-text articles excluded (n = 29):

a) Population target (n = 5)
b) Focus of study (n = 12)
c) Study outcomes (n = 10)
d) Behavioral/health-related studies (n = 2)

**The PRISMA 2020 statement: an updated guideline for reporting systematic reviews [38]**

Table 3 Characteristics of studies included

| **Authors (Year)** | **Methods** | **Population** | **Model/Content** | **Delivery methods** | **Setting** | **Key Outcome** |
| --- | --- | --- | --- | --- | --- | --- |
| Baron, Deborah, et al.  (2020) [23] | Interviews | 13 AGYW aged 16–24 years | Peer-led and activity-based empowerment clubs.    Inter-linking themes, delivered included Gender Roles and Social Norms; Power and Control; Sexual and Reproductive Health, and Empowerment. | Four 90-min sessions that study participants attended once a month on average. One interviewer facilitated club sessions. Additional open-ended club sessions were offered monthly for continued support following the conclusion of the curriculum. | Clinics, college campuses, and the community.    South Africa & Tanzania | Increased self-esteem and self-efficacy, reduced isolation, and greater insight into gender-based violence and strategies to address it. Safe space for sharing problems (such as relationship conflict and PrEP stigma) and found interactive exercises helpful in improving partner communication. |
| Christian, Canice, et al. (2023)  [31] | Mixed methods | 24 AGYW aged15–24 years | Youth club implementing the Empowerment and Livelihood for Adolescents (ELA) curriculum.    Life-skills curriculum, and Health topics education. Other activities included vocational training, indoor and outdoor sports, and mobilizing community support. | Three times per week, over six months, with trained local peer mentors. Curriculum covered one topic per club meeting. | Clinics and community.    Uganda | Integration of HIV prevention services into an established ELA curriculum at mentor-led youth clubs in rural Uganda was feasible. PrEP uptake increased among sexually active AGYW. |
| Manda, Wanangwa Chimwaza, et al.  (2021) [20] | Interviews | 23 Very young adolescents (VYA) aged 12–14 years. 18 of the girls were in primary school, 5 were out of school. 15 were living with parents, 5 with single parents and 3 with relatives. | Go Girl clubs.    Participants were trained in various skills relating to their sexual and reproductive health, clubs served as a safe space for AGYW to learn how to prevent HIV including improved access to key health services, education support, social skills, asset building, and economic strengthening. | Young women called club facilitators used a DREAMS tool kit which is a form of curriculum to train the participants. | Community    Malawi | Desire to learn about SRH was as a motivation for joining the clubs. A change of gender roles and norms at home. A decline in child abusive practices at home. Improved life skills and social networks. Economic Empowerment of guardians facilitated the support of out-of-schools girls to go back to school. Increased knowledge on sexual health issues. |
| Roberts, Sarah T., et al. (2023)[29] | Mixed methods | 103 AGYW aged 17-24 years. | Empowerment-based support club -Tu'Washindi Intervention    Curricula covered PrEP information, PrEP disclosure, undisclosed PrEP use, healthy relationships, GBV and PrEP use, unstructured session (topics of participants’ choice), unstructured session, and future goals. | Eight-session empowerment-based support club, delivered over 6 months. | Community    Kenya | Those who were not using PrEP at enrolment were more likely to initiate PrEP. Participants taking PrEP had more days with device openings. Tu'Washindi intervention showed promise in promoting PrEP uptake and adherence among AGYW without concomitant increases in intimate partner violence (IPV), but adherence remained suboptimal. |
| Roberts, Sarah T., et al. (2023)[28] | Mixed methods | 247 HIV-negative AGYW aged 16–21 | In-person and online peer support clubs or peer buddies    Information domain included the knowledge to counter misconceptions about PrEP, the motivation domain included self-worth and the desire for counsellors’ approval, and the behavioural skills and self-efficacy domain included disclosure, garnering social support and overcoming rumours, all key challenges that participants had to navigate to successfully adhere. | Adherence support included monthly counselling sessions with drug-level feedback (DLF) plus optional daily short message service (SMS) reminders, weekly phone or SMS check-ins, peer support clubs, and additional counselling. | Clinical setting    South Africa, Uganda, and Zimbabwe | REACH strategies supported adherence by providing information about HIV and PrEP, motivating participants, and supporting the development of behavioural skills and self-efficacy. Effectiveness was supported by strong interpersonal relationships with counsellors; accessible support and resources; and trust in the counsellors and study products through counsellor relationships, peer-to-peer exchange and DLF. |
| Celum, Connie, et al. (2021)[26] | Quantitative methods | 451 women aged 16-25. | PrEP adherence clubs    Not described | Monthly adherence clubs. | Clinical setting    South Africa and Zimbabwe | PrEP initiation was high. Over half of study participants persisted with PrEP through month 12. Most of young African women had detectable TFV-DP levels through month 6 with one-fifth having high adherence. Drug level feedback in the first 3 months of PrEP use did not increase the proportion with high adherence at month 6. HIV incidence was 1% in the cohort with 39% prevalence of curable STIs and moderate PrEP adherence. |
| Cassidy, Tali, et al. (2021) [27] | Mixed methods | 18-25 year old women | PrEP diva    Peer support through fun group discussions. | Gatherings were held on Saturdays. In total, seven events took place, approximately three/year. Gatherings were a way of introducing participants to peers and encouraging peer support through fun group discussions held outside the clinic setting led by study staff. | Clinic and community setting    South Africa | Majority of participants had low TFV-DP levels. The remaining 33% maintained high adherence. Forgetting to take or travel with pills were barriers. Disclosure to friends and family opened dialogue around sex, and helped to educate others about PrEP. Sex with multiple partners, and sex without a condom, declined. Participants credited their PrEP experience with changing their behaviour. |
| Velloza, Jennifer, et al. (2022) [24] | Mixed methods | 18 and 25 years AGYW | PrEP Smart    The topics were informed by participants, public holidays, and issues trending on social media and the news. | WhatsApp groups included 25–50 participants each. At enrolment, AGYW randomized to WhatsApp were entered into one of the study WhatsApp groups and the group facilitator virtually introduced her. Each group was led by a staff facilitator and a young woman who had experience in taking PrEP. Groups were also given the option to meet in person, for about one to two hours approximately quarterly, to stimulate group cohesion. | Clinical setting    South Africa | Not yet reported (Study Protocol) |
| Kennedy et al.   (2018)[25] | Quantitative methods | 15-17 year-olds | Making proud choices (MPC) and attention-matched comparison curriculum (General Health Program)    MPC focuses on HIV/AIDS-related knowledge, peer norms, sexual attitudes, and sexual behaviors. The comparator (General Health Comparison Curriculum) focused on health information on malaria, TB, worm infestations and HIV/STI knowledge. | 8 sessions led by trained health educators | Community centres, churches, youth-serving organizations    Liberia | Adapted HIV/STI program had positive effects on knowledge, sexual refusal and condom use self-efficacy, condom negotiation self-efficacy, positive condom attitudes, parental communication about sex, and negative condom attitudes over time. |
| Kumbani et al. (2023)[21] | Quantitative methods | 13-19 (Youth) and 20 or older (Adults) | Mzake peer group    Covers HIV transmission and prevention facts basic reproductive and sexuality information, prevention and treatment of other STIs, testing and treatment as prevention, partner communications, correct condom use, & engaging in community wide HIV prevention. | Six sessions, led by eight pairs of co-facilitators, each leading four groups (two adult and two youth groups). Followed by a celebration with neighbours which includes participant presentations on what they have learnt and receipt of certificates. | A community setting in a rural district    Malawi | The majority answered correctly in the intervention group than the control group at Time 2. The difference was not significant at Time 3. The HIV/PMTCT Knowledge Index scores increased over time for both groups, with the intervention group having a significantly higher increase. The intervention was highly effective in increasing knowledge among youth, but not for adults. Mzake was effective in increasing HIV prevention knowledge when delivered by community members. |
| Mash et al. (2012)[30] | Quantitative methods | 12-19 years (Youth) | Peer education programme (Fikelela: Agents of Change)    Focusing on values and relationships, exploring teenage pregnancy, HIV, sexual coercion, drugs & alcohol.  Also focusing on improving communication between parents and children and to help parents understand the challenges their children face in terms of issues of risky behaviour. | 20 life skills sessions. Each session lasted for 90 min and ran over a 12-month period. Presented by peer educators, while facilitators assisted with preparation of the sessions, logistics and group discipline. Three sessions were also held with parents and led by peer educators. | Community setting. At Anglican Churches    South Africa | The programme was successful at increasing condom usage and postponing sexual debut. There was no difference in secondary abstinence or with the number of partners. |
